# Spontaneous Adrenal Hemorrhage with Mild Hypoadrenalism in a Patient Anticoagulated with Apixaban for Antiphospholipid Syndrome: A Case Report and Literature Review

**DOI:** 10.1155/2022/6538800

**Published:** 2022-11-30

**Authors:** Jia Wei Tan, Anant Shukla, Jiun-Ruey Hu, Sachin K. Majumdar

**Affiliations:** ^1^Department of Internal Medicine, Yale New Haven Health, Bridgeport Hospital, Bridgeport, CT, USA; ^2^Section of Cardiovascular Medicine, Department of Internal Medicine, Yale School of Medicine, New Haven, CT 06510, USA; ^3^Department of Endocrinology, Yale New Haven Health, Bridgeport Hospital, Bridgeport, CT, USA

## Abstract

**Background:**

Adrenal hemorrhage (AH) is a serious endocrine complication of antiphospholipid syndrome (APLS). *Case Presentation*. We report a 45-year-old man who presented with several deep venous thromboses and was initially treated with apixaban, who later developed bilateral AH. Laboratory findings were consistent with cortisol deficiency yet preserved aldosterone physiology. He was diagnosed with APLS and treated with warfarin. After 8 months of follow-up, he remained on cortisol replacement with no evidence of recovery. We reviewed PubMed/MEDLINE indexed articles from 1950 to 2022 for cases of AH in APLS patients on anticoagulation. Six cases of patients on direct oral anticoagulants (DOACs) were reported. *Discussion*. The unique vasculature of the adrenal glands creates a “functional vascular dam” in the zona reticularis, which is susceptible to thrombosis in situ and hemorrhage. DOACs may further increase the risk of AH.

**Conclusion:**

Depending on the degree of adrenal involvement in AH, patients can present with partial or complete primary adrenal insufficiency. More data are needed to characterize adrenal function after AH, and the safety of DOAC versus warfarin in patients with APLS warrants further studies.

## 1. Introduction

Primary adrenal insufficiency (PAI) is a rare and life-threatening complication of antiphospholipid syndrome (APLS). The pathophysiology of PAI is considered due to thrombosis from the underlying coagulopathy, with possible transformation into hemorrhagic infarction [1]. The destruction of the adrenal cortex secondary to thrombosis and/or hemorrhage can progress rapidly; however, the degree to which it may affect adrenal function can vary.

The use of a new generation of direct-acting oral anticoagulants (DOACs) has largely replaced warfarin in the management of hypercoagulable state. DOACs have predictable anticoagulation effects and do not require dose monitoring, unlike warfarin. The convenience of DOACs leads to a surge in their use. However, to date, there is not an adequately powered randomized control trial establishing DOAC safety in APLS.

Our aim is to present a case of AH in APLS on DOAC and review the existing literature to inform subsequent management.

## 2. Case Report

### 2.1. Case Presentation

A 45-year-old man without significant past medical history presented with a left lower extremity deep vein thrombosis (DVT) and pulmonary embolism (PE). He had no personal or family history of previous trauma, bleeding, or clotting events. He was discharged on apixaban.

**Figure 1:**
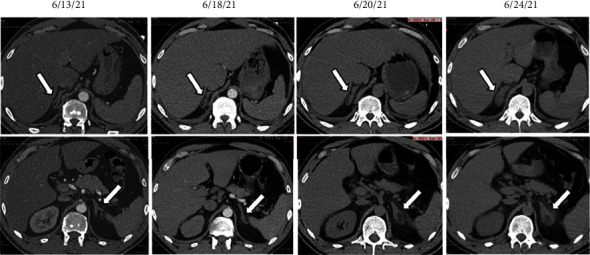
Evolution of the right (first row) and left (second row) adrenal gland enlargement.

Three days later, he was readmitted with bilateral flank pain. Abdominal CT (Figure 1) showed bilateral adrenal gland edema. He was hemodynamically stable. Due to persistent right flank pain and concern for ileus, an abdominal CT was repeated revealing enlarging adrenal glands concerning for AH (Figure 1). He subsequently complained of asthenia. Further tests revealed a reduced morning cortisol of 1.4 *μ*g/dL (reference (ref): 6.0–18.4 *μ*g/dL) and an elevated morning ACTH of 149 pg/mL (ref: 7.2–63.3 pg/mL). Plasma dehydroepiandrosterone sulphate (DHEA-S) was reduced at 39 mcg/dL (ref: 70–495 mcg/dL), and epinephrine and norepinephrine were undetectable. Plasma aldosterone in the morning was undetectable and renin activity was 0.76 ng/ml/h (ref: 0.25–5.82 ng/mL/h). He was started on stress dose steroids and discharged on maintenance hydrocortisone without reinitiating anticoagulation.

Two weeks later, he represented with a right lower extremity DVT. The APLS panel that was sent from prior admission resulted and revealed an anti-beta2 glycoprotein 1 IgG of 201 U/ml (ref: <7.0 U/mL) and anti-cardiolipin IgG of 56.4 GPL (ref: 0.0–23 GPL). He had a dilute Russell's viper venom (dRVV) normalized ratio of 3.08 (ref: ≤1.20) and silica clotting time (SCT) normalized ratio of 3.06 (ref: ≤ 1.20). Partial thrombin time (PTT) 1 : 1 mixing studies were performed. PTT at 0 seconds (s) was 36.2 s (ref: 22.0–35.0 s) and 38.7 s after 60 s, indicating the presence of lupus anticoagulant. Anti-nuclear antibody (ANA) titer was 1 : 40, and the anti-double stranded DNA antibody test was negative. Laboratory workup for other causes of thrombosis, including inherited and acquired thrombophilias, anatomical vascular obstruction, paroxysmal nocturnal hemoglobinuria (PNH), heparin-induced thrombocytopenia (HIT), and myeloproliferative neoplasms (MPN), was negative.

Warfarin was started with an international normalized ratio (INR) target range of 2-3. Hydrocortisone was continued at a total daily dose of 30 mg. Since discharge, with up to 8 months of follow-up, his morning cortisol levels prior to taking hydrocortisone have been low at 4.4–4.9 *μ*g/dL and ACTH has remained high at 92–137 pg/mL, but aldosterone has been variable at <1 to 9 ng/dL, and renin has been normal at 0.35 ng/mL/h, while DHEA-S remains low. This suggests that what transpired was a microthrombi-associated intra-adrenal hemorrhage involving the zona fasciculata and possibly reticularis with preservation of the glomerulosa.

### 2.2. Methods

We reviewed all publications that reported the development of AH from 1989 to 2022. We searched MEDLINE and PubMed with the following phrases: “adrenal insufficiency,” “adrenal hemorrhage,” “addison disease,” “antiphospholipid syndrome,” “APLS,” “novel oral anticoagulants,” “direct-acting oral anticoagulants,” “rivaroxaban,” “apixaban,” “edoxaban,” “dabigatran,” “coumadin,” “warfarin,” “vitamin K antagonist oral anticoagulants,” and “heparin.” References of retrieved articles were also examined for the key terms. Cases were analyzed with specific reference to patient demographics, clinical, laboratory, treatment, and outcome. Reports of non-APLS patients were excluded from the analysis.

### 2.3. Results

At the time of this writing, we found 8 cases [2–9] of AH in patients with APLS on anticoagulation (Table 1). Six out of 8 patients were on DOACs. The remaining 2 cases of AH were patients on warfarin, and their presenting INRs were supratherapeutic. All of the 8 patients were discharged on warfarin. Six out of 8 patients (75%) of these patients required corticosteroids and aldosterone at discharge. Long-term adrenal function is unknown.

## 3. Discussion

Adrenal hemorrhage (AH) is rare, with an estimated 15% mortality rate [10]. Risk factors for AH include anticoagulation therapy, recent surgery, tumor metastasis, corticotropin stimulation, adrenal tumor, physiological stress (trauma and burns), severe sepsis, and APLS [1].

The incidence of adrenal insufficiency in APLS is 0.4% [11]. In a series of 69 APLS patients with adrenal involvement who underwent CT and/or MRI, 57% had AH, whereas 10% had adrenal infarction, and 10% had adrenal enlargement. Adrenal involvement was bilateral in 77% of the patients [1].

Although APLS is a hypercoagulable state, biopsy (autopsy in 16 patients) in 22 APLS patients with adrenal insufficiency revealed hemorrhagic infarction with vessel thrombosis as the most frequent finding, present in 55% of patients. This is followed by adrenal hemorrhage in 27%, adrenal infarction in 5%, and normal appearance in 9% of patients [1]. These findings support the notion that in most of the cases of PAI in APLS, the primary event is adrenal vein thrombosis followed by hemorrhagic transformation.

**Figure 2:**
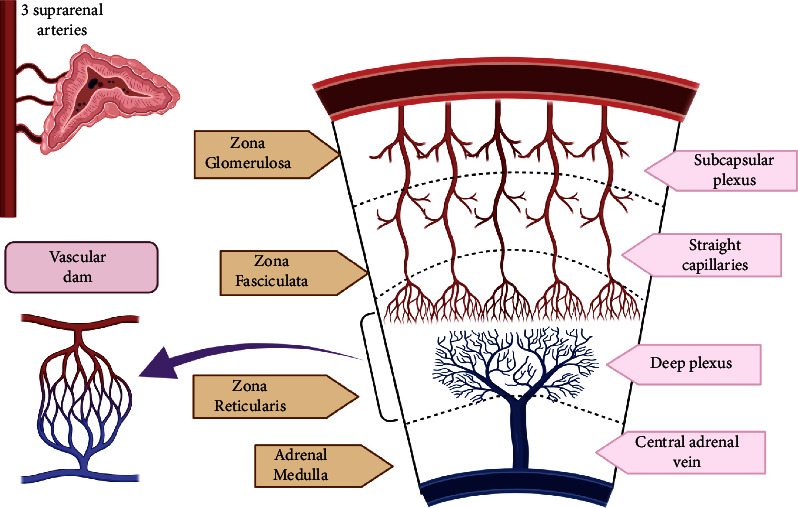
Adrenal gland circulation illustrating the abrupt transition from multiple arteries into capillary plexus, forming the “vascular dam” that increases risk of thrombotic hemorrhagic infarction.

The central pathophysiology of adrenal vein thrombosis is based on its unique vascular anatomy (Figure 2). The adrenal gland is highly vascular, with three main arteries but only one vein. The adrenal arteries branch into capillaries that form a vascular plexus around the zona reticularis. The abrupt transition of the artery-capillary plexus is referred to as a “vascular dam.” Venous stasis and hypercoagulability in this region can promote thrombosis, infarction, and subsequent hemorrhage. The capillary plexus eventually empties into medullary sinusoids that form a single central vein.

Additionally, the musculature of the vein is arranged eccentrically and composed of thick longitudinal muscle bundles. This favors the formation of thrombi in pockets of local stasis emerging from their contraction. The zona reticularis may be more severely affected compared to zona glomerulosa and fasciculata that are located further away from the vascular dam.

In our case, the diagnosis of adrenal hemorrhage was discovered on CT imaging incidentally showing adrenal congestion with evidence of mild hemorrhage. The diffuse thickening of the glands and adrenal enlargement has been reported as a precursor of adrenal hemorrhage [10, 12–14]. A high index of suspicion is needed, and any clinical indicators of acute adrenal insufficiency should prompt immediate measurement of cortisol and ACTH. Additional laboratory tests may be helpful to support the diagnosis of adrenal insufficiency and include DHEAS, aldosterone, and renin activity. It is not clear what the role of catecholamine measurement would be in these circumstances, but epinephrine production may be most noticeable as it is secreted from the medulla and depends on intra-adrenal cortisol for its synthesis. Norepinephrine may be of less value since it is also produced extra adrenally. What remains uncertain is whether recovery of adrenal function is possible in cases such as ours where there is evidence of partially preserved adrenal function.

The choice of anticoagulant in APLS may be relevant to the risk of developing AH, yet only a few case reports are available and thus one can only hypothesize that warfarin may confer less risk of AH in comparison to DOACs. It has also been reported that APLS patients developed catastrophic APLS (CAPS) after switching to DOAC from warfarin [15, 16]. Notably, they were all triple-positive APLS. AH also occurred in non-APLS patients taking DOACs [17–19].

Currently, the evidence for secondary prevention of thrombotic events in patients with APLS using DOACs is mixed [20]. Our patient had triple-positive APLS developed VTE during DOAC treatment. The risk of thrombosis with DOACs use may be stratified by the patient's APLS antibody status. In a recent retrospective study, the rate of venous thromboembolism or bleeding events with DOAC (apixaban or rivaroxaban) use among single-positive APLS patients is not significantly different from those treated with warfarin [21]. The European League Against Rheumatism (EULAR) suggests against DOAC use in triple-positive APLS [22]. Warfarin should remain the as the reference anticoagulation until further evidence regarding the safety of DOACs in APLS is available.

There is a lack of information on the course of adrenal recovery when AH occurs, particularly when there is evidence of partial preservation of function, when aldosterone appears preserved with mild to moderate deficiencies in cortisol, adrenal androgens, and possibly catecholamines. Therefore, close follow-up with periodic assessment of the HPA axis and empiric steroid replacement therapy is reasonable, and long-term outcome reporting should be encouraged to obtain prognostic information.

## 4. Conclusion

This case highlights that hypoadrenalism can be one of the complications of APLS, and the risk of AH might be heightened with DOAC use. Patients may have partially preserved adrenal function depending on the extent of adrenal involvement in AH, and biochemical and imaging evaluation is important for diagnostic and perhaps prognostic purposes. Hormonal replacement should be guided by clinical and biochemical findings as is typically done for the management of adrenal insufficiency with periodic assessment for recovery. Until further evidence is available for DOAC use in APLS, warfarin should be the first-line anticoagulation treatment.

## Figures and Tables

**Table 1:** Case reports of patients with adrenal hemorrhage while on anticoagulation for APLS.

Reference	Comuth et al. [2]	Lavoipierre [3]	Arosemena et al. [4]	Sanford et al. [5]	Bakhsh et al. [6]	DiCenso et al. [7]	Aneke and Gunendran [8]	Godfrey et al. [9]
Gender	Female	Male	Male	Male	Female	Male	Female	Female
Age (years)	63	47	46	42	40	46	53	68
Triple positive	Yes	Yes	NA	NA	NA	NA	NA	NA
Prior warfarin use	No	No	No	Yes	No	Yes	Current	Current
Type of DOAC	Rivaroxaban	Apixaban	Rivaroxaban	Apixaban	Rivaroxaban	Rivaroxaban	Warfarin	Warfarin
Duration of AC prior to admission	5 months	2 months	Few months	1 month	2 weeks	Unknown	7 years	Unknown
Clinical manifestations	LUQ abdominal pain, vomiting	Abdominal pain, livedo reticularis	Abdominal pain, nausea, weight loss	Fever, right flank pain	Severe RUQ pain, nausea	Abdominal pain, syncope	Nausea, vomiting, fever, rigors, R flank pain	Bilateral loin pain, vomiting, fever, confusion
Laboratory findings	K of 4.2 mmol/L and Na of 137 mmol/L		Na of 121 mmol/L, K of 5.4 mmol/L	NA	Renal function test reported normal	Na of 121 mmol/L, K of 5.5 mmol/L, random cortisol of 0.8 mcg/dL (22 nmol/L)	Na of 122, cortisol of 1.4 mcg/dL (40 nmol/L)	Na of 132, cortisol of 1.7 mcg/dL (48 nmol/L)
CT findings	Dilatation of both adrenal glands	Bilateral adrenal hematomas	Bilateral adrenal hemorrhage	Right-sided adrenal hemorrhage	Enlargement of the right adrenal gland	Thickening of the adrenal glands	Bilateral adrenal hemorrhage	Bilateral heterogenous adrenal pathology
MRI findings	Unknown	Unknown	T1 signal in the region of bilateral adrenal glands	Unknown	Unknown	T1 hyper-intensity of the bilateral adrenal glands	No	No
ACTH stimulation	Positive	Positive	NA	NA	NA	NA	NA	NA
Treatment	Methylpred 40 mg IV-switched to HC (dose/duration unknown)	HC + fludrocortisone	HC 50 mg q 6 hr + fludrocortisone 0.1 mg daily	Unknown	Methylpred 1.5 mg/kg	HC 50 mg IV every 8 hours	IV HC	IV HC 100 mg q 6 h->switched to oral HC + fludrocortisone
Medication upon discharge	Unknown	HC + fludrocortisone	HC 15 mg in AM and 10 mg in PM fludro 0.1 mg daily	Unknown	Unknown	HC + fludrocortisone	Oral HC-reducing doses to reach target dose: 10 mg AM, 5 mg noon, 5 mg night	HC + fludrocortisone
Anticoagulation upon discharge	Dalteparin 15.000 IE daily-switched to warfarin after a month (unknown target INR)	Warfarin	Warfarin (unknown target INR)	Warfarin (target INR 2.5–3.5)	Warfarin (target INR 2–2.5)	Unknown	Fondaparinux-switched back to warfarin after 5 days	Unknown

N/A: not applicable, DOAC: direct oral anticoagulant, Na: sodium, K: potassium, LUQ: left upper quadrant, Methylpred: methylprednisolone, HC: hydrocortisone, and IV: intravenous.

## Data Availability

No data were used to support this study.

## Abbreviations

AH: Adrenal hemorrhage
PAI: Primary adrenal insufficiency
MPN: Myeloproliferative neoplasms.
DOAC: Direct oral anticoagulant
APLS: Antiphospholipid syndrome
CAPS: Catastrophic antiphospholipid syndrome
EULAR: European league against rheumatism
HPA: Hypothalamus pituitary axis
DVT: Deep vein thrombosis
PE: Pulmonary embolism
ACTH: Adrenocorticotropic hormone
